# Efficient expression of self-complementary AAV in ganglion cells of the ex vivo primate retina

**Published:** 2009-12-16

**Authors:** Rajeshwari D. Koilkonda, William W. Hauswirth, John Guy

**Affiliations:** 1 Bascom Palmer Eye Institute, University of Miami, Miller School of Medicine, Miami, FL; 2 McKnight Vision Research Center, University of Miami, Miller School of Medicine, Miami, FL; 3 University of Florida, College of Medicine, Gainesville, FL

## Abstract

**Purpose:**

To evaluate the efficiency of self-complementary adeno-associated virus (scAAV)-mediated gene expression of green fluorescent protein (GFP) or the allotopic human ND4 subunit of complex I in ganglion cells of the primate retina.

**Methods:**

ScAAV2 containing the cDNA encoding the humanized GFP or allotopic ND4 subunit of complex I under the control of the cytomegalovirus (CMV) immediate early gene enhancer and short chicken beta-actin promoter-exon1-intron1 (CBA) was injected into the vitreous cavity of five primate eyes after enucleation. Following incubation in standard Dulbecco's Modified Eagle Medium (DMEM) culture media overnight at 37 °C with 5% CO_2_, retinal flat mounts were probed with monoclonal GFP or FLAG antibodies overnight followed by counterstaining with anti-mouse IgG conjugated to cy2. For identification of retinal ganglion cells (RGCs), the retinal whole mounts were also stained with a Brn3a or Thy1.2 (protein expressed in RGCs. domain) antibody, then counterstained with cy3 or cy2. Immunofluorescence and colocalization were assessed using confocal microscopy. Quantitative analysis of GFP, ND4FLAG, Brn3a, or Thy1.2 expressing cells was performed using Image J software.

**Results:**

While the endogenous fluorescence of GFP was seen in a few retinal cells, GFP and ND4FLAG immunofluorescence was plentiful. The immunosignals were restricted to the inner retina and colocalized to slightly more than half of all cells expressing Brn3a or Thy1.2, suggesting efficient expression in RGCs.

**Conclusions:**

Our findings suggest that the hybrid CMV enhancer-CΒA promoter can play an efficient role in targeting primate RGCs following intravitreal gene delivery using the scAAV2 vector. Donated ex vivo primate eyes may serve as a model system for testing RGC expression before in vivo intravitreal injections of this and perhaps other AAV serotypes.

## Introduction

Studies evaluating ganglion cell and optic nerve expression using the adeno-associated virus (AAV) vector for gene delivery have mostly been performed in cultured human cells [1,2] or in rodent species [3,4]. For the most part, these studies have used the chicken beta-actin promoter to drive transgene expression. This promoter and the AAV vector have been successfully used for photoreceptor gene expression when delivered by subretinal injections [5]. Most reports that have described AAV-mediated gene delivery in nonhuman primates have used subretinal injections of AAV [6-11]. One of these also studied intravitreal injections, but did not demonstrate expression of the transgene (VEGF) in retinal ganglion cells (RGCs) [7]. We were unable to find published data showing that intravitreal injections of AAV target RGCs of nonhuman primates.

Last year Merigan and coworkers [12] presented data showing that intravitreal injections of standard single-stranded (ss) AAV serotype 2 (ssAAV2) using the chicken β-actin (CBA) promoter did not drive expression of GFP in RGCs, but rather in foveal cones. Since intravitreal gene delivery to RGCs is central to our mitochondrial gene therapy studies of patients with Leber Hereditary Optic Neuropathy (LHON) [13] and optic neuritis [14], we evaluated the efficiency of the CBA promoter used successfully in our rodent studies [3,13,15,16] to drive reporter green fluorescent protein (GFP) gene expression in the primate eye and further to characterize whether expressed protein was localized to RGCs.

## Methods

### Viral preparation

The humanized gene for GFP (GeneBank U50963) was inserted into a self-complementary AAV (scAAV) vector (regulated by the 381 bp cytomegalovirus (CMV) immediate early gene enhancer /578 bp CBA promoter-exon1-intron1). Plasmids were amplified and purified by cesium chloride gradient centrifugation and then packaged into AAV-2 capsids by transfection into human 293 cells using standard procedures [17]. Vector preparations were produced by the plasmid cotransfection method. The crude iodixanol fraction [17], as described, was further purified and concentrated by column chromatography on a 5 ml HiTrap Q Sepharose column using a Pharmacia AKTA FPLC system (Amersham Biosciences, Piscataway, NJ). The vector was eluted from the column using 215 mM NaCl, pH 8.0, and the recombinant adeno-associated virus (rAAV) peak collected. Vector-containing fractions were then concentrated and buffer exchanged in Alcon Balanced Salt Solution (Bss; Alcon Laboratories, Fort Worth, TX) with 0.014% Tween-20, using a Biomax 100 K concentrator (Millipore, Billerica, MA). Vector was then titered for DNase-resistant vector genomes by real-time PCR relative to a standard [17]. Finally, the purity of the vector was validated by silver-stained sodium dodecyl sulfate–PAGE, assayed for sterility and lack of endotoxin (endotoxin-PTS rapid endotoxin detection kit, Charles River), and then aliquoted and stored at −80 °C. The resultant rAAV-packaged GFP or allotopic human ND4FLAG virus preparation contained 10^11^ to 10^12^ vector genome-containing particles per milliliter.

### AAV and flat-mount retina preparation

The anterior segments were carefully removed. Next 10 μl of scAAV-CBA-GFP was injected into the vitreous of three eyes that had been enucleated from two rhesus macaque by Dr. Jonathan Horton (University of California, San Francisco, CA) approximately 12 h earlier. We then injected 10 μl of scAAV-CBA-ND4FLAG into two donor cynomolgus monkey eyes, received from Dr. Jean-Marie Parel (Bascom Palmer Eye Institute), within 20–30 min of enucleation. After incubation in standard Dulbecco’s Modified Eagle Medium culture media overnight at 37 °C with 5% CO_2_, retinas were gently separated from the eyecups. Flat-mounted retinas were prepared and laid out with the RGC layer facing upward. The tissue was blocked and permeabilized with 20% goat serum, 0.2% Triton X-100 in PBS (10×, 1.37 M NaCl, 0.027 M KCl, and 0.119 M Phosphate Buffer, pH 7.4) for 30 min at room temperature. This was followed by immunostaining with mouse monoclonal GFP or FLAG primary antibody for 12 h at 4°C. Primary antibody solution is routinely contained in 10% goat serum and 0.2% Triton X-100 in PBS, pH 7.4. The tissues were then washed and incubated in secondary antibody with the fluorolink cy2 (AAV-GFP-inoculated eyes) or cy3 (AAV-ND4FLAG-inoculated eyes) conjugated goat anti-mouse IgG at room temperature for 2 h in PBS containing 10% goat serum and 0.2% Triton X-100. The tissue was washed with PBS and observed with the Zeiss Axiovert 200M Fluorescence/Live cell Imaging Microscope (Carl Zeiss Inc., Thornwood, NY). Further, for identifying the retinal cells that were positive for GFP or FLAG, the retinal whole mounts were washed again, permeabilized in the aforedescribed same manner and incubated in primary antibody Brn3a or Thy1.2 in PBS, pH 7.4 with 10% goat serum and 0.2% Triton X-100, at 4 °C for 12 h. The retinal tissues were washed (washed twice and done at room temperature-RT) and incubated in secondary antibody cy3 (AAV-GFP inoculated eyes) or cy2 (AAV-ND4FLAG inoculated eyes) conjugated goat anti-mouse IgG at room temperature for 2 h in PBS containing 10% goat serum and 0.2% Triton X-100. The tissue was washed with PBS and observed for fluorescence with a Leica TCS SP5 confocal microscope. The retinas were then cryoprotected in sucrose buffers and snap frozen. Cryo-sections counterstained with DAPI were examined for immunofluorescence. Velocity software was used to assess colocalization of GFP or ND4FLAG to Brn3a- or Thy1.2-expressing pixels and Image J software was used for quantitative analysis of GFP- or ND4FLAG-expressing cells to Brn3a- or Thy1.2-positive RGCs.

## Results

### scAAV-GFP

**Figure 1:**
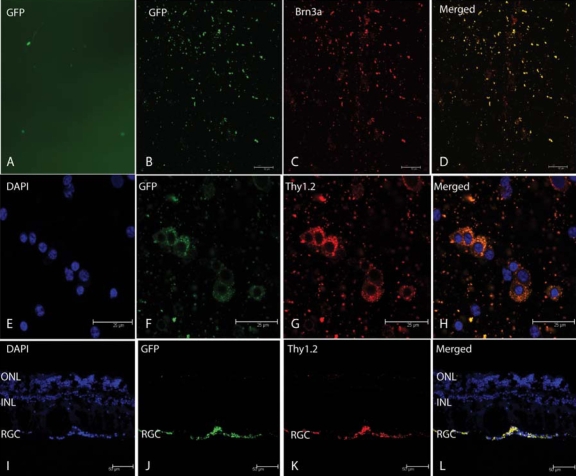
Fluorescence Microscopy GFP. Retinal flat mounts inoculated with scAAV-GFP revealed some green fluorescent protein (GFP) positive cells (**A**). Confocal microscopy revealed many more GFP positive cells by immunofluorescence (**B**). RGCs labeled with the Brn3a antibody (**C**) and merged images revealed GFP expressing cells co-localized exclusively with those expressing Brn3a (**D**). Higher power confocal micrographs of scAAV-GFP injected eyes show nuclei labeled by DAPI (**E**). Cells expressing GFP (**F**) or Thy1.2 (**G**) co-localized (**H**). Cryo-sectioned retinas of scAAV-GFP injected eyes counterstained with DAPI revealed the nuclei of cells in the retinal ganglion cell layer (RGC), inner nuclear layer (INL) or outer nuclear layer (ONL; **I**). GFP immunofluorescence is seen exclusively in the ganglion cell layer (**J**) whose RGCs were labeled by Thy1.2 (**K**). Colocalization of GFP and Thy1.2 cells is shown in the merged panel (**L**).

**Figure 2:**
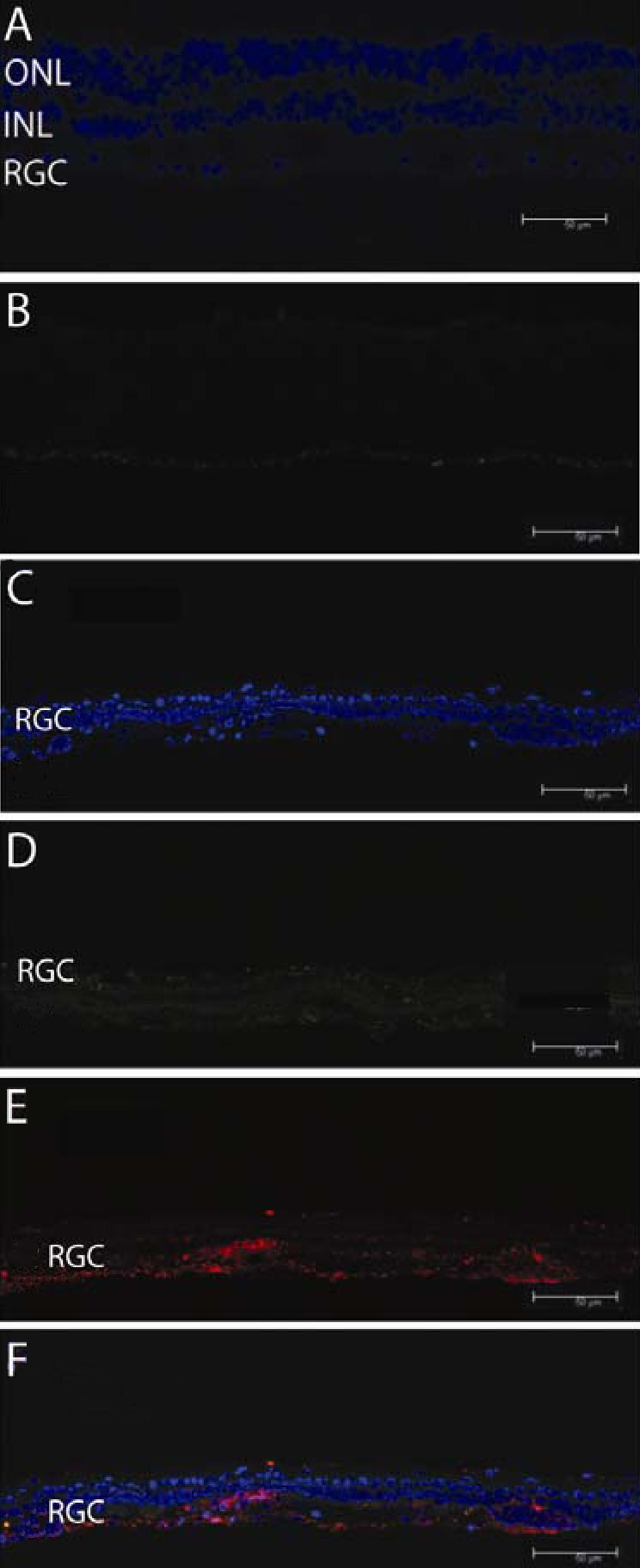
Fluorescence microscopy controls. Retinal cross-sections counterstained with DAPI (**A**) without the primary anti-green fluorescent protein (GFP) antibody that were counterstained with the secondary antibody showed no GFP labeling (**B**). DAPI stained RGC layer (**C**) from specimen double-labeled for GFP (**D**) and Thy1.2 are shown (**E**). The merged image of panels **C-E** shows Thy1.2 RGCs without GFP positivity (**F**). Abbreviations: ONL represents outer nuclear layer, INL represents inner nuclear layer, RGC represents retinal ganglion cell. Scale bar equals to 50 µm.

Fluorescence microscopy of retinal flat mounts inoculated with scAAV-GFP revealed GFP-positive cells (Figure 1A). Confocal microscopy of retinal flat mounts from eyes injected with scAAV-GFP revealed that GFP immunofluorescence was plentiful (Figure 1B). RGCs labeled with the Brn3a antibody are shown in Figure 1C. The merged images revealed that GFP-expressing cells colocalized exclusively with those expressing Brn3a (Figure 1D), thus confirming that GFP expression occurred in primate RGCs. As indicated by the absence of cells that were labeled only green in panels 1D, we were unable to visualize any GFP-expressing cells that did not also express Brn3a. In addition, as indicated by many red labeled cells, not all Brn3a-positive RGCs expressed GFP. Higher power confocal micrographs of scAAV-GFP-injected eyes showed nuclei labeled by DAPI (Figure 1E). Cells expressing GFP (Figure 1F) or Thy1.2 (Figure 1G) colocalized (Figure 1H). Cryosectioned retinas of scAAV-GFP-injected eyes counterstained with DAPI revealed the nuclei of cells in the RGC layer, inner nuclear layer, or outer nuclear layer (Figure 1I). The GFP immunofluorescence was seen exclusively in the ganglion cell layer (Figure 1J), in which RGCs were labeled by Thy1.2 (Figure 1K). Colocalization of GFP and Thy1.2 cells is shown in the merged panel (Figure 1L). Control cross-sections counterstained with DAPI (Figure 2A) without the primary anti-GFP antibody that were counterstained with the secondary antibody showed no GFP signal (Figure 2B). DAPI stained the RGC layer (Figure 2C) of a specimen that was double-labeled for GFP (Figure 2D) and Thy1.2 (Figure 2E). The merged panel (Figure 2F) shows Thy1.2-labeled RGCs that were negative for GFP.

### scAAV-ND4FLAG

**Figure 3:**
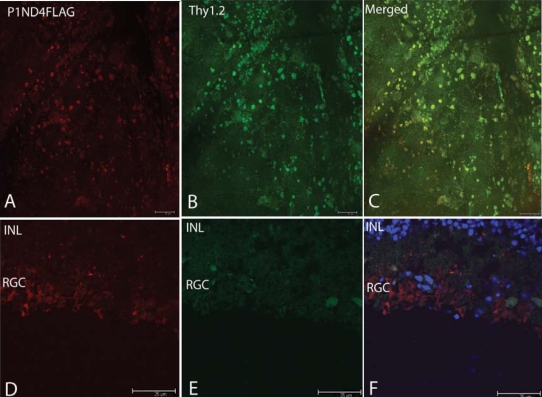
Fluorescence microscopy ND4FLAG. Confocal microscopy of a retinal flat mount from an eye injected with the scAAV-ND4FLAG packaged in VP3 capsids with the tyrosine to phenylalanine transition at position 444 revealed many cells were labeled with the anti-FLAG antibody (**A**). RGCs expressing Thy1.2 (**B**) colocalized with ND4FLAG expressing cells are shown (**C**). Confocal imaging of longitudinal cross-sections of the retina revealed ND4FLAG immunofluorescence within the RGC layer (**D**). Immunofluorescent labeling of Thy1.2 positive cells with cy2 was somewhat fading by the time of confocal imaging of cryosectioned retina (**E**). A merged panel counterstained with DAPI shows ND4FLAG immunofluorescence within the RGC layer (**F**). Abbreviations: INL represents inner nuclear layer, RGC represents retinal ganglion cell. Scale bar equals to 50 µm.

Confocal microscopy of a retinal flat mount from an eye injected with the scAAV-ND4FLAG packaged in VP3 capsids with the tyrosine to phenylalanine transition at amino acid position 444 reveals many cells were labeled with the anti-FLAG antibody (Figure 3A). RGCs expressing Thy1.2 are shown in Figure 3B. Colocalization of ND4FLAG- and Thy1.2-expressing cells are shown in the merged panel (Figure 3C), thus indicating that most cells expressing the ND4FLAG fusion protein were RGCs. Confocal imaging of longitudinal cross-sections of the retina revealed ND4FLAG immunofluorescence within the RGC layer (Figure 3D). Immunofluorescent labeling of Thy1.2 cells with cy2 was somewhat fading by the time of confocal imaging of cryosectioned retina (Figure 3E). However, the merged panel also counterstained with DAPI showed ND4FLAG immunofluorescence within the RGC layer (Figure 3F). The opposite eye injected with scAAV-ND4FLAG but packaged with the standard VP3 capsids with intact tyrosine residues showed no labeling with the anti-FLAG antibody (data not shown).

### Efficiency of GFP and ND4FLAG expression

We compared the number of cells that were immunopositive for either GFP or ND4FLAG to the number of ganglion cells as identified by Brn3a or Thy1.2 immunoreactivity. There were 486 GFP-positive cells/40 mm^2 ^ relative to 805 Brn3a-positive RGCs /40 mm^2 ^(n=2) and 174 GFP-positive cells/40 mm^2 ^ relative to 293 Thy1.2-positive cells/40 mm^2^ (n=1). Similarly, there were 225 FLAG-positive cells/40 mm^2 ^ relative to 293 Thy1.2-positive cells/40 mm^2^ (n=1). Thus the efficiency of allotopic and xenotypic expression in the ex vivo primate was possibly as high as about 60%.

## Discussion

Last year Merigan and coworkers [12] presented data suggesting that in vivo intravitreal injections of ssAAV serotype 2 using the chicken beta-actin promoter did not efficiently drive expression of GFP in RGCs, but resulted in GFP expression in foveal cones of the primate eye. We show here that when injected into the vitreous of the ex vivo primate eye, the hybrid CMV enhancer-CΒA promoter, successfully used in rodents, efficiently drives GFP expression in ganglion cells of the nonhuman primate retina. These are the very cells affected by LHON [18]. The ssAAV2 has been used safely in a phase I human ocular gene therapy trial [19]. Newer AAV vectors such as the scAAV that contains complementary strands for delivery [20-22] or other AAVs with mutations in the capsid proteins designed to reduce cellular AAV degradation [23,24] that may provide the promise of higher levels of efficiency of transgene expression have not been tested in the primate retina until now. We showed here that the allotopic ND4 subunit of complex I delivered by a scAAV2 is expressed in primate RGCs with an efficiency comparable to that of GFP delivered by an ssAAV2 in our rodents.

The robust expression of transgene in RGCs only 12 h after vector injection deserves comment. We used a scAAV vector rather than the standard single-stranded version. Deletion of 25 bases in the 3′ region of the forward terminal repeat of AAV results in generation of an AAV containing both complementary strands [21,22]. It is generation of the complementary strand of ssAAV that is believed to be the rate-limiting step in expression from standard ssAAV vectors and leads to the several week delay in transgene expression typically seen from ssAAV vectors in vivo. A scAAV like the one used here resulted in transgene expression of GFP within days after injection in rodents [25,26]. Why would such rapidity of expression be important clinically? Increasing the speed of ND4 gene expression may be relevant to the treatment of LHON patients who have acute visual loss that must be treated quickly [1,2,16]. We found GFP expressed as early as four days after intraocular injection of mice with the scAAV-GFP, while taking approximately two weeks with ssAAV-GFP in a parallel, equal titer experiment. With scAAV-GFP the efficiency of RGC labeling was not only faster but it was superior to ssAAV. Rescue of optic neuropathy in the rodents [27] suggests that allotopic ND4 gene therapy may be effective in LHON patients with the G11778A mitochondrial DNA mutation. Our expression studies using the AAV vector in the primate eye suggest that the AAV cassette will result in transgene expression in humans.

Recently it has been shown that AAV vectors with capsid mutations may further increase transgene expression in RGCs [25]. These vectors were constructed based on the finding that a key limiting factor in AAV transduction efficiency is phosphorylation of tyrosine residues exposed on the vector capsid surface. Normally this leads to ubiquination and proteosomal degradation of vector before its genome can be delivered to the cell nucleus [28]. Petrs-Silva and coworkers [25] have been testing this idea in the mouse retina using Tyr-Phe (Y-F) mutations at several surface tyrosine residues in AAV serotype 2, 8, and 9 vectors. Using a self-complementary CBA-GFP vector insert, they quantified a 10–15-fold enhancement in RGC GFP expression compared to the corresponding wild-type parent for each serotype, including AAV2 used here, four weeks after intravitreal injection of equal titers of AAV vectors. While one of these mutants (tyrosine to phenylalanine substitution at amino acid 444 of the VP3 capsid) produced the highest levels of GFP fluorescence, the intravitreal injections of high titer virus also resulted in labeling of retinal cells other than RGCs. Reducing viral titers confined GFP expression to RGCs. We showed here that scAAV-ND4FLAG packaged with this mutant VP3 had an efficiency of RGC expression in the primate retina of 60% comparable to that of GFP.

For testing approaches to augmenting potency applicable to optic neuropathy, we believe that donated ex vivo primate eyes may serve as a platform for modulating transgene expression in RGCs as a prelude to in vivo studies of living nonhuman primates and eventually human gene therapy trials. Rose and coworkers have also found that the cultured primate retina survives days after enucleation and that it is an extremely useful model system for evaluations of RGC and axonal regeneration [29]. It is highly likely that optic neuropathy patients selected for gene therapy trials will have mild or moderate degrees of RGC or axonal injury from LHON or optic neuritis, with the axotomized retina employed here probably representing the most severe form of injury [30].
